# Deep brain stimulation for obsessive-compulsive disorder and treatment-resistant depression: systematic review

**DOI:** 10.1186/1756-0500-3-60

**Published:** 2010-03-04

**Authors:** Shaheen E Lakhan, Enoch Callaway

**Affiliations:** 1 Global Neuroscience Initiative Foundation, Los Angeles, CA, USA

## Abstract

**Background:**

In spite of advances in psychotherapy and pharmacotherapy, there are still a significant number of patients with depression and obsessive-compulsive disorder that are not aided by either intervention. Although still in the experimental stage, deep brain stimulation (DBS) offers many advantages over other physically-invasive procedures as a treatment for these psychiatric disorders. The purpose of this study is to systematically review reports on clinical trials of DBS for obsessive-compulsive disorder (OCD) and treatment-resistant depression (TRD). Locations for stimulation, success rates and effects of the stimulation on brain metabolism are noted when available. The first observation of the effects of DBS on OCD and TRD came in the course of using DBS to treat movement disorders. Reports of changes in OCD and depression during such studies are reviewed with particular attention to electrode locations and associated adverse events; although these reports were adventitious observations rather than planned. Subsequent studies have been guided by more precise theories of structures involved in DBS and OICD. This study suggests stimulation sites and prognostic indicators for DBS. We also briefly review tractography, a relatively new procedure that holds great promise for the further development of DBS.

**Methods:**

Articles were retrieved from MEDLINE via PubMed. Relevant references in retrieved articles were followed up. We included all articles reporting on studies of patients selected for having OCD or TRD. Adequacy of the selected studies was evaluated by the Jadad scale. Evaluation criteria included: number of patients, use of recognized psychiatric rating scales, and use of brain blood flow measurements. Success rates classified as "improved" or "recovered" were recorded. Studies of DBS for movement disorders were included if they reported coincidental relief of depression or reduction in OCD. Most of the studies involved small numbers of subjects so individual studies were reviewed.

**Results:**

While the number of cases was small, these were extremely treatment-resistant patients. While not everyone responded, about half the patients did show dramatic improvement. Associated adverse events were generally trivial in younger psychiatric patients but often severe in older movement disorder patients. The procedures differed from study to study, and the numbers of patients was usually too small to do meaningful statistics or make valid inferences as to who will respond to treatment.

**Conclusions:**

DBS is considered a promising technique for OCD and TRD. Outstanding questions about patient selection and electrode placement can probably be resolved by (a) larger studies, (b) genetic studies and (c) imaging studies (MRI, fMRI, PET, and tractography).

## Background

In the middle of the twentieth century, the lesioning of areas of the brain was discovered to be an effective treatment for certain movement disorders. Unfortunately, there were damaging side effects as a result of these lesions. In the 1980s, it was determined that the same effects could be accomplished by stimulating the tissue with electricity. DBS was approved by the FDA as a treatment for movement disorders in 2002. Although still in the research stage, DBS appears to also be a major advance in the treatment of obsessive compulsive disorder (OCD) and treatment resistant depression (TRD).

Electrophysiology and modern imaging allow the very precise placement of electrodes. Response can be optimized by changing location and adjusting stimulus site parameters. Since the patient cannot detect the simulation, periods of active stimulation and sham (zero voltage) can be alternated to provide single or double-blind controls.

Stimulation usually has a pulse width around 60 microseconds delivered at 130 Hz, although there are variations on these parameters depending on the target. Bipolar and monopolar electrodes have been used; monopolar electrodes were contact cathode and case anode. After the sites and parameters for DBS are selected, the stimulator is placed under the skin, usually below the clavicle, and connecting wires are run under the skin to the stimulating electrodes in the brain. Although the sites for stimulation vary, they basically fall into two groups: a basal ganglia group and a cingulate gyrus group.

The primary purpose of this article is to summarize all clinical trials of DBS in OCD and TRD, with particular attention to outcomes and electrode locations.

## Methods

### Searching

We conducted a comprehensive search using MEDLINE/PubMed in May 2009, using different combinations of the following MeSH and free text terms: deep brain stimulation, DBS, obsessive-compulsive disorder, OCD, depression. Searches were restricted to human studies, clinical trials, and reviews. Reference lists from retrieved reports were reviewed for additional relevant studies. Unpublished data were not sought and there were no language restrictions. [See Additional file 1 for a Quality of Reporting of Meta-analyses (QUOROM) statement checklist.]

### Selection

We included human studies that assessed the efficacy of DBS on psychiatric symptoms for OCD, TRD, or both. We accepted papers with only one case if they included some estimate of efficacy. In general, discussions of ethical issues were not reviewed. Articles discussing only physical measurements (e.g., cerebral blood flow) or transient effects (e.g., smiling) were rejected from the main analysis but some were referenced in the discussion.

### Validity assessment

Each of the primary studies were rated using the Jadad scale [1], but see Berger 2006 for critical commentary [2]. This scale gives one point for each of the following, (a) randomization, (b) randomization appropriate and well-described, (c) double-blind, (d) double-blind appropriate and well-described, and (e) withdrawals and dropouts described. No studies were excluded for poor scores. Psychiatric rating scales and cerebral blood flow studies were noted when used. Improvement of these severely ill patients by chance has a very low probability and that the nature of DBS lends itself to using the patient as its own control, by simply turning off the stimulator, as there is usually no sensation from DBS. This is almost universally taken advantage of in past studies. On the negative side, many studies were small, and actual techniques so complex that between-study comparison was ineffective.

### Data abstraction

Data were extracted independently by the authors and any disagreements were resolved by consensus.

### Analysis

Due to the differences in outcome measures used in the trials, a quantitative analysis of the data was deemed inappropriate. A qualitative summary of the data was consequently completed.

## Results

### Flow of included studies

**Figure 1:**
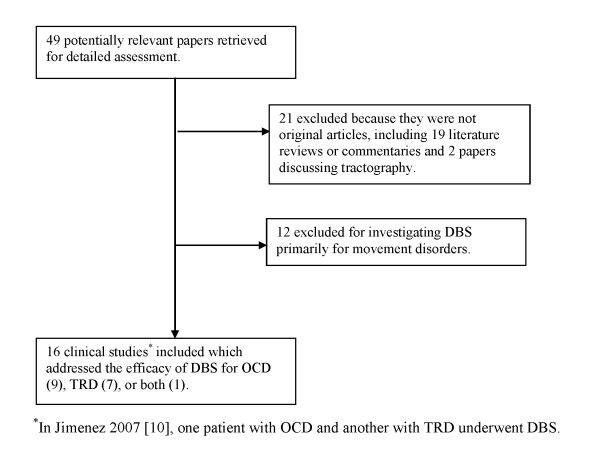
**Flow diagram of included studies**.

Electronic searches found 49 studies that were potentially relevant to the present systematic review. Of these, 16 met the inclusion/exclusion criteria (see Figure 1 for a flow diagram). Of the 33 that did not meet the criteria, 12 were excluded from the main review because they investigated DBS primarily for movement disorders with only incidental observation of psychological side effects on depressive or OCD symptoms. Papers that did not explicitly deal with the psychiatric symptoms of patients were excluded. However, 21 of the excluded papers are listed as reviews and some are also cited in the discussion. Papers that were mainly discussions of philosophical and ethical issues were not reviewed at all.

We found reference to a relatively new procedure called tractography [3]. Tractography is a magnetic resonance imaging (MRI) technique that shows relationships in the human brain between a "seed" location and those structures to which the seed has functional connections. This is highly relevant for future selection of DBS targets and is briefly described in the discussion section.

### Study characteristics

Sixteen studies published between 1999 and 2009 were selected for review: nine for OCD [4-12], seven for TRD [10,13-18], and one for both disorders [19] (in Jimenez et al., 2007 [10], one patient with OCD and another with TRD underwent DBS). These studies included a total of 42 patients with OCD who were treated with DBS, 67 patients with TRD, and one with both OCD and TRD. Length of study ranged from 3 to 39 months. All availed themselves of the use of patients as their own controls by turning stimulation on and off (single-blind), but Table 1 indicates those that had the clinical observations made blind (double-blind). Randomization of patient selection was never used although random selection of active/sham sessions was.

**Table 1:** Analysis of 16 trials reporting on the efficacy of DBS treatment for OCD, TRD, or both.

	Study	Jadad score	Double blind	No. of patients (started/finished)	Final observation period (months)	Cerebral blood flow	Clinical scales	DBS location	Adverse effects*	Patients improved	Patients recovered
**OCD**	Nuttin 1999 [4]	3	Yes	6/4	21	fMRI, PET	POMS	AL/IC		3 (75%)	

	Gabriels 2003 [5]	0	No	3/3	33-39		BPRS, POMS, Y-BOCS	AL/IC		2 (66%)	

	Nuttin 2003 [6]	4	Yes	6/4	21	fMRI, PET	CGI, Y-BOCS	AL/IC, DMNT		3 (50%)	

	Sturm 2003 [7]	0	No	4/4	30-34	fMRI, PET		Right NA		4 (100%)	

	Abelson 2005 [8]	3	No	4/4	10	PET	GAF, HDRS	AL/IC	Mild dizziness	3 (75%)	1 (25%)

	Greenberg 2006 [9]	4	Yes	10/8	36		HDRS, Y-BOCS	AL/IC, VC/C	Seizure, hypomania, relapse with battery failure	2 (25%)	4 (50%)

	Jimenez 2007** [10]	0	Yes	1/1			GAF	ITP		1 (100%)	1 (100%)

	Mallet 2008 [11]	4	Yes	18/168	3		GAF, CGI MADRAS, MDRS, MINI, Y-BOCS	STN	15 major including a brain hemorrhage, 22 minor adverse events	10 (62%)	4 (25%)

	Nuttin 2008 [12]	4	Yes	6/6	21	PET	CGI, Y-BOCS	AL/IC		3 (75%)	

**TRD**	Jimenez 2005 [13]	0	No	1/1	24		HDRS	ITP			1 (100%)

	Mayberg 2005 [14]	3	Yes	6/6	6	PET	CGI, HDRS, MADRS, PANAS	CG			4 (66%)

	Schlapfer 2005 [15]	4	Yes	3/3		PET					3 (100%)

	Jimenez 2007** [10]	0	No	1/1			HDRS, GAS	ITP			1 (100%)

	Lozano 2008 [16]	4	Yes	20/20	12	PET	BAI, BDI, CGI, HDRS	CG		12 (60%)	7 (35%)

	Malone 2009 [17]	4	No	15/15	15		CGI, GAF, HDRS, MADRS	VC/CS			6 (40%)

	Wang 2009 [18]	0	Yes	21/21***			HDRS	STN			

**OCD and TRD**	Aouizerate 2009 [19]	0		1/1	15		HDRS, Y-BOCS	LCa, NA		1 (100%)	

AL/IC, anterior limbs of internal capsule; BAI, Beck Anxiety Inventory; BDI, Beck Depression Inventory; BPRS, Brief Psychiatric Rating Scale; CG, cingulate gyrus; CGI, Clinical Global Impressions; DMNT, dorsa-medial nucleus of the thalamus; GAF, Global Assessment of Function; GAS, Global Assessment Scale; ITP, inferior thalamic peduncle; LCa, limbic caudate; HDRS, Hamilton Depression Rating Scale; MADRS, Montgomery-Asberg Depression Rating Scale; MDRS, Mattis Dementia Rating Scale; MINI, Mini International Neuropsychiatric Interview; NA, nucleus accumbens; PANAS, Positive and Negative Affects Scales; PET, positron emission tomography; PMOS, Profile of Mood States; STN, subthalamic nucleus; VC/CS, ventral capsule/ventral striatum; Y-BOCS, Yale-Brown Obsessive-Compulsive Scale.

Blank cells indicate that there were no data on that subject in the paper.

*Other than minor surgical effects.

**In this study, one patient with OCD and another with TRD underwent DBS.

***Patients on anti-Parkinson drugs were controls.

There are a number of generally accepted measures of well-being, depression, and OCD, and we have indicated the ones used in the major studies. For general well-being there are the Global Assessment Scale (GAS), Clinical Global Impressions (CGI) scale, Global Assessment of Function (GAF), and Positive and Negative Affects Scales (PANAS). For OCD, the standard is the Yale-Brown Obsessive-Compulsive Scale (Y-BOCS). For TRD, the standard assessments include the Hamilton Depression Rating Scale (HDRS), the Montgomery-Asberg Depression Rating Scale (MADRAS), Beck Depression Inventory (BDI), and Beck Anxiety Inventory (BAI). There is also the Brief Psychiatric Rating Scale (BPRS), Profile of Mood States (PMOS), and Self-Rating Depression Scale (SDS) used in some studies.

See Table 1 for specific characteristics of each reviewed study. The table shows primary psychiatric diagnoses, Jadad scores, numbers of subjects starting and numbers completing, durations of studies, methods of cerebral blood flow measurement (if assessed), clinical ratings scales used, locations of DBS, adverse events reported (if more than minor surgical squeals), numbers and percent improved, numbers and percent recovered, and whether ratings were done double-blind.

### Reports on patients with OCD

Nuttin et al.,1999 [4] (POMS). There were six patients with severe OCD. Quadripolar electrodes were implanted into the anterior limb of the internal capsule (AL/IC). Four patients continued for 21 months. One patient was unchanged and three were much improved after 21 months. Stimulation resulted in changes in regional activity as measured by functional MRI (fMRI) and positron emission tomography (PET) scan.

Gabriels et al., 2003 [5] (BPRS, POMS, Y-BOCS). Three severe OCD patients were treated with DBS to the anterior limbs of the internal capsules. One patient with somatoform symptoms showed no improvement. The two others essentially recovered. Their gains endured with minimal adverse events.

Nuttin et al., 2003 [6] (CGI, Y-BOCS). Six patients were implanted with quadripolar electrodes in AL/IC. Four were evaluated double-blind. One was unchanged and three greatly improved after 21 months. The non-responder was preoccupied with body hair but failed to fulfill the criteria for body dimorphic disorder.

Strum et al., 2003 [7]. Four treatment-resistant OCD patients with co-morbid severe anxiety disorder received DBS to the right nucleus accumbens. Bilateral stimuli did not improve results. Three of the four had total remissions over 24 to 30 months.

Abelson et al., 2005 [8] (GAF, HDRS). Using hardware developed for the treatment of movement disorder, four patients with chronic intractable OCD were treated with leads placed bilaterally in the anterior limbs of their anterior capsules. They received random four-week blocks of stimulus on and off. Dramatic improvement was seen in one patient and a second showed moderate improvement. The unimproved patient failed to show the regional cerebral blood flow (rCBF) changes seen in patients who responded to treatment, referred to in this discussion as responders. Residual depression left one OCD responder unable to function normally.

Greenberg et al., 2006 [9] (HDRS, Y-BOCS). Ten OCD patients with severe treatment-resistant OCD had quadrapolar leads implanted rostral to the anterior commisure extending into the ventral capsule and ventral commisure. Patients were followed for 36 months. Four patients had improvement greater than 34% based on the Yale-Brown Obsessive Compulsive Scale. Two patients had declines between 25 and 35%. Incidental mention is made of improvement of depression. Side effects included an asymptomatic hemorrhage, seizure, superficial infection, worsening of symptoms when DBS stopped due to battery failure, and transient hypomanic symptoms.

Jimenez et al., 2007 [10] (GAF). See also under TRD. A female with depression for 23 years and a male with obsessive-compulsive disorder for nine years had stereotactic implantation of electrodes in the inferior thalamic peduncle. Using the GAF scale, both cases showed improvement.

Mallet et al., 2008 [11] (GAF, GAI, MADRAS, Y-BOCS, CGI, MDRS, Mattis Dementia Rating Scale, Mini International Neuropsychiatric Interview). There were 16 patients in a multi-center study of severe OCD. They used randomized crossover design with 10 months subthalamic or sham stimulation. There were significant improvements in OCD with active stimulation, but there were 15 major (including a brain hemorrhage) and 23 minor adverse events. Four of the patients recovered, using a Y-BOCS of 6 or less as an indicator of recovery. It was noted that there was no improvement in depression although hypomania was one of the adverse events.

Nuttin et al., 2008 [12] (CGI, Y-BOCS). DBS electrodes were inserted into the anterior limbs of the internal capsule of six severe OCD patients. Stimuli caused an increase in fMRI signal, particularly in the pons. PET scans at three months showed lower frontal metabolism. Symptoms were much improved in three patients and unchanged in one by 21 months.

### Reports of patients with TRD

Jimenez et al., 2005 [13] (HDRS). The patient was a 48-year-old woman with TRD. Tetrapolar-stimulating electrodes were implanted bilaterally in the inferior thalamic peduncles. Stimulation was 130 Hz, .45 ms pulse width and 2.5 volts. At 24 months, the woman had a sustained recovery with minimal adverse events.

Mayberg et al., 2005 [14] (CGI, HDRS, MADRAS, PANAS). Six patients with severe chronic TRD were treated. On stimulation of white matter adjacent to the subgenual cingulate gyrus, all subjects reported acute effects that included calmness, heightened awareness, and increased visual acuity. Chronic treatment resulted in sustained remissions in four of the six patients at the six-month evaluation mark with associated improvements in performance and sleep. Antidepressant effects were associated with a reduction in rCBF in local and down-stream limbic and cortical sites. Adverse events were minor and included skin infections in two patients and erosion over the hardware. There was a suggestion that non-responders were atypical depressives with late onsets.

Schlaepfer et al., 2005 [15]. Three patients with TRD were implanted with bilateral DBS electrodes in the nucleus accumbens. Stimulation parameters were modified in a double-blind manner, and clinical ratings assessed at each modification. Brain metabolism was assessed one week before and one week after stimulation. Clinical ratings improved in all three patients when the stimulator was on, and worsened in all three patients when the stimulator was off. Effects were observed immediately, and no side effects occurred in any of the patients. Using PET, significant changes in brain metabolism as a function of the stimulation in frontostriatal networks were observed.

Jimenez et al., 2007 [10] (GAS, HDRS). See also under OCD. One patient with inferior thalamic peduncle DBS recovered.

Lozano et al., 2008 [16] (BDI, BAI, BDI, CGI, HDRS). Twenty patients with TRD underwent serial assessments before and after DBS of the subcallosal cingulate gyrus. One month after surgery, 35% of patients were responders and 10% were in remission. Six months after surgery, 60% were responders and 35% were in remission. Benefits were maintained at 12 months. DBS was associated with changes in the metabolic activity of cortical and limbic circuits implicated in the pathogenesis of depression. Adverse effects were trivial and transient.

Malone et al., 2009 [17] (CGI, GAF, HDRS, MADRAS). Fifteen TRD patients were given ventral caudate-ventral striatum DBS. At six months, nearly 50% of patients responded and 20% achieved remission. At the last examination (six months to four years), there were about the same number of responders and 40% of patients were in remission.

Wang et al., 2009 [18] (HDRS, SDS). Using a subthalamic nucleus (STN)-DBS group (n = 27) and an anti-Parkinson's medication control group, evaluation of depression and motor function was performed six times using SDS and HDRS. Depression decreased notably within six months postoperatively. The mean value of the bilateral voltages was correlated with SDS and HDRS scores (P < 0.05). The improvement in motor symptoms resulting from STN-DBS can improve depression in Parkinson's disease (PD) patients, but its long-term effects were unremarkable. Within the treatment range, the mean value of bilateral voltages correlated with severity of depression in PD patients.

### Reports of patients with both OCD and TRD

Aouizerate et al., 2009 [19] (HDRS, Y-BOCS). The first case was also reported in 2004 [20]. Two patients with intractable depression and OCD were treated with DBS. Caudate stimulation relieved the OCD symptoms while nucleus accumbens-stimulation improved depressive symptoms.

## Discussion

For as long as the brain has been seen as the site of mental activity, it has followed that altering brain function should be implemented to treat mental illness. Second generation antidepressants and psychotherapy are currently the least invasive ways of affecting brain function [21,22] but they leave too many patients only partially improved, and have proved completely ineffective for some. Estimates of treatment unresponsiveness are unreliable, but 30% to 40% patients with depression and OCD probably become treatment failures. For these patients, techniques like DBS provide a promising treatment alternative.

### Limitations of this study

Currently, the chief problem is that current studies are too small to support valid inferences about patient selection and choice of stimulation locations. In the preceding sections, relevant studies have been summarized since the sparse data precludes combining them for meta-analysis.

There is a wealth of data in pain and movement disorder literature which has not been analyzed in detail. However, the survey given above suggests that DBS is not entirely benign; the reports of suicides and psychoses are disturbing. Nevertheless, it is difficult to draw general conclusions only from studies which gave incidental attention to OCD and TRD in DBS for movement disorder reports.

The basal ganglia are complex, with some subdivisions small and tightly packed together. In spite of the sophisticated imaging and electrophysiology techniques, localizations often remain less than exact.

Last year, Elsevier launched a new journal titled "Brain Stimulation." Among publications in other journals, the following references were selected as being of potential interest to the reader: [23-40]. Some of these have also been referred to in the discussion.

### Anatomy of DBS sites

The thalamic/capsular area is favored for treatment of OCD. A useful review of the basal ganglia areas is to be found in the paper by Kopell [41]. The various locations for DBS in TRD include orbito-frontal cortex, anterior cingulate gyrus, corpus striatum, globus pallidus, subgenual cingulate, ventral capsule/ventral striatum, ventral capsule/ventral commisure, nucleus accumbens, and inferior thalamic peduncle. Based on current technology and understanding of psychiatric circuitry, there seems to be two main areas where DBS can affect TRD: the cingulate white matter and the AL/IC. Nuttin [6] reported on one patient who had stimulation delivered both to the AL/IC and to the dorsa-medial nucleus of the thalamus (DMNT). Symptoms, word fluency and memory improved with AL/IC stimulation and worsened with DMNT. Stimulating the internal capsule, Okun [42] found non-mood-related responses, such as taste and smell, significantly associated with the ventral lead. Mayberg [14] found hyperactivity in the subgenual cingulate (BA25) along with hypoactivity in prefrontal (BA9/46), premotor (BA6), dorsal anterior cingulate (BA24) and anterior insula in all treatment-resistant patients. Responders had greater prefrontal hypoactivity and an area of hyperactivity in the medial frontal cortex (BA10).

### New insights into functional brain anatomy

Measurement of rCBF can be used to trace interrelationships between areas of the brain and to see if DBS has had an effect and it may also have prognostic value. Many of the areas that are supposedly affected by DBS are overactive in OCD and TRD, and certain patterns of brain metabolism may characterize some non-responders.

rCBF has traditionally been measured using PET. Radionuclides used in PET are typically isotopes with short half-lives such as carbon-11 (~20 min.), nitrogen-13 (~10 min.), oxygen-15 (~2 min.), and fluorine-18 (~110 min.). They must be delivered from a cyclotron and synthesized into organic compounds within minutes, so the procedure is limited to special facilities.

Using PET, Seminowicz et al. [36] reported on path modeling of limbic-frontal circuitry in depression. They reported that drug treatment responders and non-responders had different limbic-cortical connections, non-responders had additional limbic-subcortical abnormalities, and there were limbic-cortical and cortico-cortical differences between cognitive-behavior therapy responders and pharmacotherapy responders.

fMRI has become a reasonable and economical substitute for PET. Blood oxygen level-dependent (BOLD) refers to the MRI contrast of oxygenated with deoxygenated blood, and allows MRI measurement of rCBF.

Knowledge of specific pathways involved in OCD and TRD is obviously critical to further advances in the uses of DBS for psychiatric disorders. Tractography, also known as diffusion tensor imaging, is a relatively new procedure for determining functional connections between brain locations in living humans. Although the mathematics involved is complex, the MRI is simply used to measure the movement of oxygen along pathways from a "seed" voxel to areas that receive signals from the seed. A good discussion on it has been made by Taylor [43].

More germane to the DBS discussion, Gutman [3] has used tractographic analysis to examine the connections from seeds in the cingulate gyrus (CG) to the AL/IC. These are the two areas for DBS that are featured prominently in Table 1. They found that CG connected to the medial frontal cortex, anterior and posterior cingulate, medial temporal lobe, medial thalamus, hypothalamus, nucleus accumbens and dorsal brain stem. AL/IC projected to frontal pole, median temporal lobe, cerebellum, nucleus accumbens, thalamus, hypothalamus, and brainstem. While the two seeds were connected to some common areas, those connections seemed to be via different white matter bundles. Gutman suggested the two locations exist within separate neural networks that include common nodes. Larger studies, including both control subjects and patients, are needed for further clarification but until in vivo tract tracing in human is possible, results cannot be fully verified.

### Challenges of DBS clinical trials

The usual ideas about double blind testing and random selection are not applicable to DBS. Because of the bimodal nature of the patients' responses, and the small sizes of the samples, calculations on sample sizes for a given level of confidence must be non-parametric (for example, see Mallet [11]).

1. The patient is always his/her own control. The simulator can usually be turned off without the patient aware of the change, but sometimes there is a dramatic return of symptoms. The clinical rater is usually blind, but is the study truly double blind if turning off the stimulator precipitates suicidal ideation or obsessive pre-occupations? Randomization usually means random assignment to stimulus-first and sham-first conditions.

2. Recruitment of severe treatment resistant patients for a neurosurgical procedure has been difficult, and multi-center studies have been the only way to enroll meaningful numbers of subjects.

3. Achieving a consensus among Institutional Review Boards can be both expensive and time consuming.

4. Given the necessity of multi-center studies, one is left to assume that very complex procedures can be replicated exactly across different institutions.

As more studies are done, and if DBS becomes covered by insurance, then random selection of subjects for alternative DBS targets may become practical and parametric statistical power calculations will be feasible. Genetic studies and brain imaging could possibly improve the selection of probable responders in future.

### Ethical considerations

Many papers listed as reviews also contain comments on ethics, and the consensus is that modifying personality is not unethical. But there are other ethical considerations of DBS that need to be carefully considered. The risks and benefits of implanting electrodes in the brain must be weighed carefully as it is an invasive procedure. There also may be ethical issues involved selecting the population that receives deep brain stimulation. Would all people have equal access to treatment? If not, who would determine when a depression is severe or unresponsive enough for DBS? If it proves effective, should we allow DBS to be used for neural enhancement? These issues require comprehensive study beyond the scope of this paper.

## Conclusions

DBS is an expensive treatment and the phase for the procedure involves considerable time. It is not entirely without risks, as the adverse events described in the movement disorders suggest. OCD and TRD reports are remarkable for their lack of adverse events (other than those purely related to surgery), but that may reflect the use of younger subjects.

Table 1 provides the most compelling data in support of DBS, the number of recovered patients. These patients were severely ill individuals and all past treatment was unsuccessful. The economics of the matter would make providing such patients with expensive yet successful treatment justifiable, as the costs of repeatedly failing treatments are huge. From a humanitarian and financial viewpoint, treatment expense is not a major issue.

Thus, while still in the research stage, DBS promises to be a major advance. Modern imaging methods allows precise placement of electrodes. Electrophysiological micro-recordings from implanted electrodes before and during stimulation aid in defining the electrode locations. Measurement of rCBF before and after stimulation provides additional accuracy. Location and parameters of the stimulation can be changed to individualize and optimize treatment. Since the patient usually cannot detect whether stimulation is on or off, artificial stimulation can easily be used to deceive participants and provide a control condition. This ability to use patients as their own controls is a powerful tool for reducing placebo effects.

DBS is a very promising new development for the treatment of severe treatment-resistant depression and obsessive-compulsive disorder. So far the clinical samples are small, and some of the theoretical rationales are less than clear. Nonetheless, the results so far are very impressive, and it is certain that present shortcomings will be addressed in the near future.

## Abbreviations

AL/IC: anterior limbs of internal capsule; BAI: Beck Anxiety Inventory; BDI: Beck Depression Inventory; BOLD: blood oxygen level-dependent; BPRS: Brief Psychiatric Rating Scale; CG: cingulate gyrus; CGI: Clinical Global Impressions; DBS: deep brain stimulation; DMNT: dorsa-medial nucleus of the thalamus; EEG: electroencephalography; fMRI: functional magnetic resonance imaging; GAF: Global Assessment of Function; GAS: Global Assessment Scale; GPi: globus pallidus internal; HDRS: Hamilton Depression Rating Scale; MADRS: Montgomery-Asberg Depression Rating Scale; MRI: magnetic resonance imaging; OCD: obsessive-compulsive disorder; rCBF: regional cerebral blood flow; PANAS: Positive and Negative Affects Scales; PD: Parkinson's disease; PET: positron emission tomography; PMOS: Profile of Mood States; SDS: Self-Rating Depression Scale; STN: subthalamic nucleus; TRD: treatment-resistant depression; Y-BOCS: Yale-Brown Obsessive-Compulsive Scale.

## Competing interests

The authors declare that they have no competing interests.

## Authors' contributions

SEL and EC participated in the preparation of the manuscript. All authors read and approved the final manuscript.

## Supplementary Material

Additional file 1 **QUOROM Statement Checklist**. A PDF document showing the Quality of Reporting of Meta-analyses (QUOROM) statement checklist. Click here for file

## References

[B1] Jadad AR Moore RA Carroll D Jenkinson C Reynolds DJ Gavaghan DJ McQuay HJ Assessing the quality of reports of randomized clinical trials: is blinding necessary? Control Clin Trials 1996 17 1 12 10.1016/0197-2456(95)00134-4 8721797

[B2] Berger VW Is the Jadad score the proper evaluation of trials? J Rheumatol 2006 33 1710 1711 author reply 1711-1712 16881132

[B3] Gutman DA Holtzheimer PE Behrens TE Johansen-Berg H Mayberg HS A tractography analysis of two deep brain stimulation white matter targets for depression Biol Psychiatry 2009 65 276 282 10.1016/j.biopsych.2008.09.021 19013554 PMC4423548

[B4] Nuttin B Cosyns P Demeulemeester H Gybels J Meyerson B Electrical stimulation in anterior limbs of internal capsules in patients with obsessive-compulsive disorder Lancet 1999 354 1526 10.1016/S0140-6736(99)02376-4 10551504

[B5] Gabriels L Cosyns P Nuttin B Demeulemeester H Gybels J Deep brain stimulation for treatment-refractory obsessive-compulsive disorder: psychopathological and neuropsychological outcome in three cases Acta Psychiatr Scand 2003 107 275 282 10.1034/j.1600-0447.2003.00066.x 12662250

[B6] Nuttin BJ Gabriels LA Cosyns PR Meyerson BA Andreewitch S Sunaert SG Maes AF Dupont PJ Gybels JM Gielen F Demeulemeester HG Long-term electrical capsular stimulation in patients with obsessive-compulsive disorder Neurosurgery 2003 52 1263 1272 10.1227/01.NEU.0000064565.49299.9A 12762871

[B7] Sturm V Lenartz D Koulousakis A Treuer H Herholz K Klein JC Klosterkotter J The nucleus accumbens: a target for deep brain stimulation in obsessive-compulsive- and anxiety-disorders J Chem Neuroanat 2003 26 293 299 10.1016/j.jchemneu.2003.09.003 14729131

[B8] Abelson JL Curtis GC Sagher O Albucher RC Harrigan M Taylor SF Martis B Giordani B Deep brain stimulation for refractory obsessive-compulsive disorder Biol Psychiatry 2005 57 510 516 10.1016/j.biopsych.2004.11.042 15737666

[B9] Greenberg BD Malone DA Friehs GM Rezai AR Kubu CS Malloy PF Salloway SP Okun MS Goodman WK Rasmussen SA Three-year outcomes in deep brain stimulation for highly resistant obsessive-compulsive disorder Neuropsychopharmacology 2006 31 2384 2393 10.1038/sj.npp.1301165 16855529

[B10] Jimenez F Velasco F Salin-Pascual R Velasco M Nicolini H Velasco AL Castro G Neuromodulation of the inferior thalamic peduncle for major depression and obsessive compulsive disorder Acta Neurochir Suppl 2007 97 393 398 full_text 17691327 10.1007/978-3-211-33081-4_44

[B11] Mallet L Polosan M Jaafari N Baup N Welter ML Fontaine D du Montcel ST Yelnik J Chereau I Arbus C Subthalamic nucleus stimulation in severe obsessive-compulsive disorder N Engl J Med 2008 359 2121 2134 10.1056/NEJMoa0708514 19005196

[B12] Nuttin BJ Gabriels LA Cosyns PR Meyerson BA Andreewitch S Sunaert SG Maes AF Dupont PJ Gybels JM Gielen F Demeulemeester HG Long-term electrical capsular stimulation in patients with obsessive-compulsive disorder Neurosurgery 2008 62 966 977 10.1227/01.neu.0000333764.20575.d6 18695582

[B13] Jimenez F Velasco F Salin-Pascual R Hernandez JA Velasco M Criales JL Nicolini H A patient with a resistant major depression disorder treated with deep brain stimulation in the inferior thalamic peduncle Neurosurgery 2005 57 585 593 discussion 585-593 10.1227/01.NEU.0000170434.44335.19 16145540

[B14] Mayberg HS Lozano AM Voon V McNeely HE Seminowicz D Hamani C Schwalb JM Kennedy SH Deep brain stimulation for treatment-resistant depression Neuron 2005 45 651 660 10.1016/j.neuron.2005.02.014 15748841

[B15] Schlaepfer TE Lieb K Deep brain stimulation for treatment of refractory depression Lancet 2005 366 1420 1422 10.1016/S0140-6736(05)67582-4 16243078

[B16] Lozano AM Mayberg HS Giacobbe P Hamani C Craddock RC Kennedy SH Subcallosal cingulate gyrus deep brain stimulation for treatment-resistant depression Biol Psychiatry 2008 64 461 467 10.1016/j.biopsych.2008.05.034 18639234

[B17] Malone DA Jr Dougherty DD Rezai AR Carpenter LL Friehs GM Eskandar EN Rauch SL Rasmussen SA Machado AG Kubu CS Deep brain stimulation of the ventral capsule/ventral striatum for treatment-resistant depression Biol Psychiatry 2009 65 267 275 10.1016/j.biopsych.2008.08.029 18842257 PMC3486635

[B18] Wang X Chang C Geng N Li N Wang J Ma J Xue W Zhao W Wu H Wang P Gao G Long-term effects of bilateral deep brain stimulation of the subthalamic nucleus on depression in patients with Parkinson's disease Parkinsonism Relat Disord 2009 15 8 587 91 10.1016/j.parkreldis.2009.02.006 19403325

[B19] Aouizerate B Cuny E Bardinet E Yelnik J Martin-Guehl C Rotge JY Rougier A Bioulac B Tignol J Mallet L Distinct striatal targets in treating obsessive-compulsive disorder and major depression J Neurosurg 2009 111 4 775 9 10.3171/2009.2.JNS0881 19284243

[B20] Aouizerate B Cuny E Martin-Guehl C Guehl D Amieva H Benazzouz A Fabrigoule C Allard M Rougier A Bioulac B Deep brain stimulation of the ventral caudate nucleus in the treatment of obsessive-compulsive disorder and major depression. Case report J Neurosurg 2004 101 682 686 10.3171/jns.2004.101.4.0682 15481726

[B21] Gabbard GO Mind, brain, and personality disorders Am J Psychiatry 2005 162 648 655 10.1176/appi.ajp.162.4.648 15800133

[B22] Ressler KJ Mayberg HS Targeting abnormal neural circuits in mood and anxiety disorders: from the laboratory to the clinic Nat Neurosci 2007 10 1116 1124 10.1038/nn1944 17726478 PMC2444035

[B23] Fitzgerald P Brain stimulation techniques for the treatment of depression and other psychiatric disorders Australas Psychiatry 2008 16 183 190 10.1080/10398560701874291 18568624

[B24] Gabriels L [Neuromodulation... and then?] Tijdschr Psychiatr 2007 49 61 63 17290334

[B25] Glannon W Deep-brain stimulation for depression HEC Forum 2008 20 325 335 10.1007/s10730-008-9084-3 19130251

[B26] Kopell BH Greenberg B Rezai AR Deep brain stimulation for psychiatric disorders J Clin Neurophysiol 2004 21 51 67 10.1097/00004691-200401000-00007 15097294

[B27] Linden DE What, when, where in the brain? Exploring mental chronometry with brain imaging and electrophysiology Rev Neurosci 2007 18 159 171 17593878 10.1515/revneuro.2007.18.2.159

[B28] Lipsman N Neimat JS Lozano AM Deep brain stimulation for treatment-refractory obsessive-compulsive disorder: the search for a valid target Neurosurgery 2007 61 1 11 discussion 11-13 10.1227/01.neu.0000279719.75403.f7 17621014

[B29] Nuttin B Gybels J Cosyns P Gabriels L Meyerson B Andreewitch S Rasmussen SA Greenberg B Friehs G Rezai AR Deep brain stimulation for psychiatric disorders Neurosurg Clin N Am 2003 14 xv xvi 10.1016/S1042-3680(03)00007-X 12856486

[B30] Piallat B Chabardes S Devergnas A Torres N Allain M Barrat E Benabid AL Monophasic but not biphasic pulses induce brain tissue damage during monopolar high-frequency deep brain stimulation Neurosurgery 2009 64 156 162 discussion 162-153 10.1227/01.NEU.0000336331.88559.CF 19145164

[B31] Rauch SL Dougherty DD Malone D Rezai A Friehs G Fischman AJ Alpert NM Haber SN Stypulkowski PH Rise MT A functional neuroimaging investigation of deep brain stimulation in patients with obsessive-compulsive disorder J Neurosurg 2006 104 558 565 10.3171/jns.2006.104.4.558 16619660

[B32] Rodriguez RL Fernandez HH Haq I Okun MS Pearls in patient selection for deep brain stimulation Neurologist 2007 13 253 260 10.1097/NRL.0b013e318095a4d5 17848865

[B33] Sakas DE Panourias IG Singounas E Simpson BA Neurosurgery for psychiatric disorders: from the excision of brain tissue to the chronic electrical stimulation of neural networks Acta Neurochir Suppl 2007 97 365 374 full_text 17691325 10.1007/978-3-211-33081-4_42

[B34] Sartorius A Henn FA Deep brain stimulation of the lateral habenula in treatment resistant major depression Med Hypotheses 2007 69 1305 1308 10.1016/j.mehy.2007.03.021 17498883

[B35] Sauleau P Eusebio A Vandenberghe W Nuttin B Brown P Deep brain stimulation modulates effects of motivation in Parkinson's disease Neuroreport 2009 20 622 626 10.1097/WNR.0b013e32832aa928 19319004

[B36] Seminowicz DA Mayberg HS McIntosh AR Goldapple K Kennedy S Segal Z Limbic-frontal circuitry in major depression Neuroimage 2004 22 409 418 10.1016/j.neuroimage.2004.01.015 15110034

[B37] Trepanier LL Kumar R Lozano AM Lang AE Saint-Cyr JA Neuropsychological outcome of GPi pallidotomy and GPi or STN deep brain stimulation in Parkinson's disease Brain Cogn 2000 42 324 347 10.1006/brcg.1999.1108 10753483

[B38] Troster AI Neuropsychology of deep brain stimulation in neurology and psychiatry Front Biosci 2009 14 1857 1879 10.2741/3347 19273169

[B39] Voon V Saint-Cyr J Lozano AM Moro E Poon YY Lang AE Psychiatric symptoms in patients with Parkinson disease presenting for deep brain stimulation surgery J Neurosurg 2005 103 246 251 10.3171/jns.2005.103.2.0246 16175853

[B40] Wichmann T Delong MR Deep brain stimulation for neurologic and neuropsychiatric disorders Neuron 2006 52 197 204 10.1016/j.neuron.2006.09.022 17015236

[B41] Kopell BH Rezai AR Chang JW Vitek JL Anatomy and physiology of the basal ganglia: implications for deep brain stimulation for Parkinson's disease Mov Disord 2006 21 Suppl 14 S238 246 10.1002/mds.20958 16810674

[B42] Okun MS Mann G Foote KD Shapira NA Bowers D Springer U Knight W Martin P Goodman WK Deep brain stimulation in the internal capsule and nucleus accumbens region: responses observed during active and sham programming J Neurol Neurosurg Psychiatry 2007 78 310 314 10.1136/jnnp.2006.095315 17012341 PMC2117652

[B43] Taylor WD Hsu E Krishnan KR MacFall JR Diffusion tensor imaging: background, potential, and utility in psychiatric research Biol Psychiatry 2004 55 201 207 10.1016/j.biopsych.2003.07.001 14744459

